# Quantitative hematoma heterogeneity associated with hematoma growth in patients with early intracerebral hemorrhage

**DOI:** 10.3389/fneur.2022.999223

**Published:** 2022-10-21

**Authors:** Mingpei Zhao, Wei Huang, Shuna Huang, Fuxin Lin, Qiu He, Yan Zheng, Zhuyu Gao, Lveming Cai, Gengzhao Ye, Renlong Chen, Siying Wu, Wenhua Fang, Dengliang Wang, Yuanxiang Lin, Dezhi Kang, Lianghong Yu

**Affiliations:** ^1^Department of Neurosurgery, Neurosurgery Research Institute, The First Affiliated Hospital, Fujian Medical University, Fuzhou, China; ^2^Clinical Research and Translation Center, The First Affiliated Hospital, Fujian Medical University, Fuzhou, China; ^3^Fujian Provincial Clinical Research Center for Neurological Diseases, The First Affiliated Hospital, Fujian Medical University, Fuzhou, China; ^4^Department of Epidemiology and Health Statistics, School of Public Health, Fujian Medical University, Fuzhou, China

**Keywords:** intracerebral hemorrhage (ICH), hematoma growth, coefficient of variation (CV%), predictors, non-contrast computed tomography (NCCT), stroke

## Abstract

**Background:**

Early hematoma growth is associated with poor functional outcomes in patients with intracerebral hemorrhage (ICH). We aimed to explore whether quantitative hematoma heterogeneity in non-contrast computed tomography (NCCT) can predict early hematoma growth.

**Methods:**

We used data from the Risk Stratification and Minimally Invasive Surgery in Acute Intracerebral Hemorrhage (Risa-MIS-ICH) trial. Our study included patients with ICH with a time to baseline NCCT <12 h and a follow-up CT duration <72 h. To get a Hounsfield unit histogram and the coefficient of variation (CV) of Hounsfield units (HUs), the hematoma was segmented by software using the auto-segmentation function. Quantitative hematoma heterogeneity is represented by the CV of hematoma HUs. Multivariate logistic regression was utilized to determine hematoma growth parameters. The discriminant score predictive value was assessed using the area under the ROC curve (AUC). The best cutoff was determined using ROC curves. Hematoma growth was defined as a follow-up CT hematoma volume increase of >6 mL or a hematoma volume increase of 33% compared with the baseline NCCT.

**Results:**

A total of 158 patients were enrolled in the study, of which 31 (19.6%) had hematoma growth. The multivariate logistic regression analysis revealed that time to initial baseline CT (*P* = 0.040, odds ratio [OR]: 0.824, 95 % confidence interval [CI]: 0.686–0.991), “heterogeneous” in the density category (*P* = 0.027, odds ratio [OR]: 5.950, 95 % confidence interval [CI]: 1.228–28.828), and CV of hematoma HUs (*P* = 0.018, OR: 1.301, 95 % CI: 1.047–1.617) were independent predictors of hematoma growth. By evaluating the receiver operating characteristic curve, the CV of hematoma HUs (AUC = 0.750) has a superior predictive value for hematoma growth than for heterogeneous density (AUC = 0.638). The CV of hematoma HUs had an 18% cutoff, with a specificity of 81.9 % and a sensitivity of 58.1 %.

**Conclusion:**

The CV of hematoma HUs can serve as a quantitative hematoma heterogeneity index that predicts hematoma growth in patients with early ICH independently.

## Introduction

Spontaneous intracerebral hemorrhage is difficult to treat and continues to be a significant cause of morbidity and mortality globally (1, 2). Only one in every five survivors is self-sufficient after 6 months, with a 30-day mortality rate ranging from 30 to 40% (3, 4). Hematoma growth is associated with increased mortality and poor prognosis following intracerebral hemorrhage (5, 6). Early detection of hematoma growth can enable more aggressive treatment techniques to be implemented (7, 8). Although the computed tomography angiography (CTA) spot sign is a wellestablished predictor of hematoma formation, it is not frequently performed in many centers, particularly centers in areas with limited medical treatment (9, 10). Consequently, non-contrast computed tomography (NCCT) markers have garnered considerable interest. Originally, density and shape of a hematoma were utilized to predict hematoma growth (11). Later, other studies established the utility of NCCT in predicting hematoma growth (12–16). However, NCCT markers have several drawbacks. Numerous NCCT markers describe similar characteristics; however, there is no agreement on the appropriate image acquisition procedure, assessment, terminology, or diagnostic criteria (17). Therefore, it is essential to explore a quantitative index that can be used to anticipate the growth of hematomas based on information obtained using NCCT. The shape and density of hematomas are significantly represented by various NCCT markers. We used CT density measurement technology to quantify hematoma quantitative heterogeneity. The purpose of this study was to determine the correlation between quantitative heterogeneity and early hematoma growth.

## Materials and methods

### Study design and population

We used data from the Risk Stratification and Minimally Invasive Surgery in Acute Intracerebral Hemorrhage (Risa-MIS-ICH) trial, which was a prospective multicenter cohort study. This study was registered in ClinicalTrials.gov (No. NCT03862729). The present study utilized retrospective data from this database, from January 2015 to October 2021. Patients with the time to baseline NCCT less than 12 h and time to follow-up CT less than 72 h were included. The exclusion criteria were as follows: (i) CT at baseline was not NCCT; (ii) surgical intervention was performed before follow-up CT; and (iii) CT image quality was not optimum. The study was authorized by the Ethics Committee of the First Affiliated Hospital of Fujian Medical University (Ethical Approval Number: MRCTA, ECFAH of FMU [2018] 082-1). Furthermore, this study adhered to applicable Chinese laws, rules, and guidelines, in addition to the tenets of the Declaration of Helsinki.

### Definition of variables

Heterogeneous density of ICH was measured on a 5-point visual analog scale along with an incremental continuum. The density category of hematoma was described as “heterogeneous,” when there were at least three hypodense lesions within the dense hematoma, and “homogeneous,” when there were less than three hypodense lesions within the dense hematoma, as assessed on an axial section showing the maximum cross-sectional area of the hematoma (11, 17). The “swirl sign” was defined as an area of low or equal attenuation (compared with the attenuation of the brain parenchyma) in a high-attenuation brain hemorrhage. Areas of low or equal attenuation could vary in shape and can be circular, striated, or irregular. It could be at the edge of the hematoma (12). The “black hole sign” was defined as a relatively low-attenuation area (black hole) encased within a high-attenuation hematoma. The black hole could be round, oval, or rod-shaped, but not connected to adjacent brain tissue. The relatively low-attenuation region should have identifiable boundaries, with a difference of at least 28 HUs between the two density areas (14, 17). The “blend sign” of hematoma was defined as a relatively low-attenuation region within the hematoma mixed with an adjacent high-attenuation region. A clear border between the low-attenuation region and the adjacent high-attenuation region should be easily identifiable by the naked eye, with a difference of at least 18 HUs between the two density regions of the hematoma. The two denser zones should be easily distinguishable by direct visual inspection of the scan without image zooming (13). “Deep ICH” was described as ICH involving the thalamus, basal ganglia, internal capsule, or deep periventricular white matter, whereas “lobar ICH” was classified as ICH originating at the cortex and cortical–subcortical junction (18, 19). “Hematoma growth” was defined as an absolute growth hematoma volume of more than 6 mL or a relative growth of more than 33% the volume from baseline CT to follow-up CT within 72 h (20–22).

### Imaging analysis

**Figure 1:**
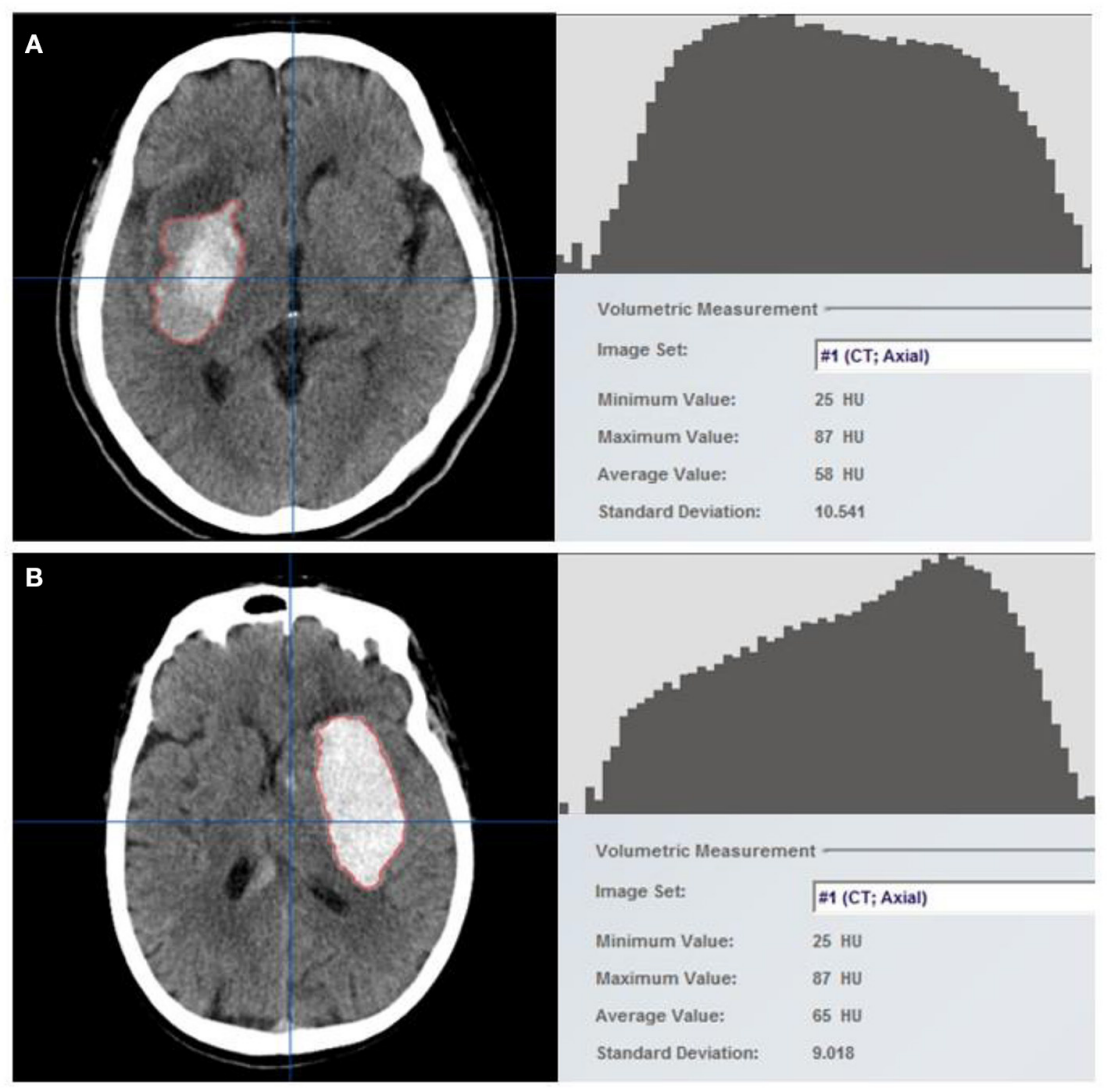
Hematomas were identified using the semi-automatic edge detection tool included in neuro-navigation workstation software **(left)**. The auto-segmentation function was used to process the region of interest (ROI), which constituted the entire hematoma, to obtain the Hounsfield unit histogram and density-related parameters **(right)**. Two example cases with (**A**) and without (**B**) hematoma growth are shown. Hounsfield units (HUs) are generally discrete in patients with hematoma growth, with a higher coefficient of variation (CV) of 18%, but are concentrated in patients without hematoma growth, with a CV of 14%.

Initial and follow-up CT scans are performed using normal clinical techniques. For future processing and evaluation, all image data were archived in the Digital Imaging and Communications in Medicine (DICOM) format. To determine the volume and density of the hematoma, two independent researchers (Mingpei Zhao and Wei Huang) examined the baseline NCCT markers of 158 patients by workstation software (iPlan 3.0, Brainlab, Feldkirchen, Germany). The researchers were unaware of the patients' clinical history and follow-up CT findings. Hematomas were detected layer by layer on the axial section using a semi-automatic edge detection method. The region of interest (ROI) included the entire hematoma and was processed using the auto-segmentation function to obtain the histogram of HU, the mean of HU, and the coefficient of variation (CV) of HU (Figure 1). Detailed processing is shown in Supplementary Image. The follow-up CT images were processed in the same manner, and two stroke neurologists independently reviewed all measurement results (Liang-Hong Yu and Fu-Xin Lin). The CV of hematoma HU represented the heterogeneity of hematomas.

### Statistical analysis

Categorical variables were described as percentages, and the chi-square test or Fisher's test was used to determine the distribution differences across groups. Continuous variables with a normal distribution were presented as means and standard deviations, compared using a two-tailed Student's *t*-test. The median (25^th^−75^th^ quartile) of skewed data was used to compare them using the Mann–Whitney U test. We utilized univariate analysis to identify potential relevant determinants of hematoma growth. We then used multivariate logistic regression to determine the independent determinants of hematoma growth. In the multivariate analysis, factors with *P* < 0.05 in the univariate analysis and those known to be associated with hematoma growth as confounders were included. The optimal cutoff was determined using the receiver operating characteristic (ROC) curve analysis, and the predictive value of the discriminant score was determined using the area under the receiver operating characteristic (AUC) curve analysis. SPSS version 26.0 (SPSS Inc., Chicago, Illinois, USA) and R version 4.1.0 (“R” foundation for statistical computing, Vienna, Austria) were used for analysis. Two-tailed *P*-values were reported, and *P* < 0.05 was considered statistically significant.

## Results

### Patient characteristics

**Figure 2:**
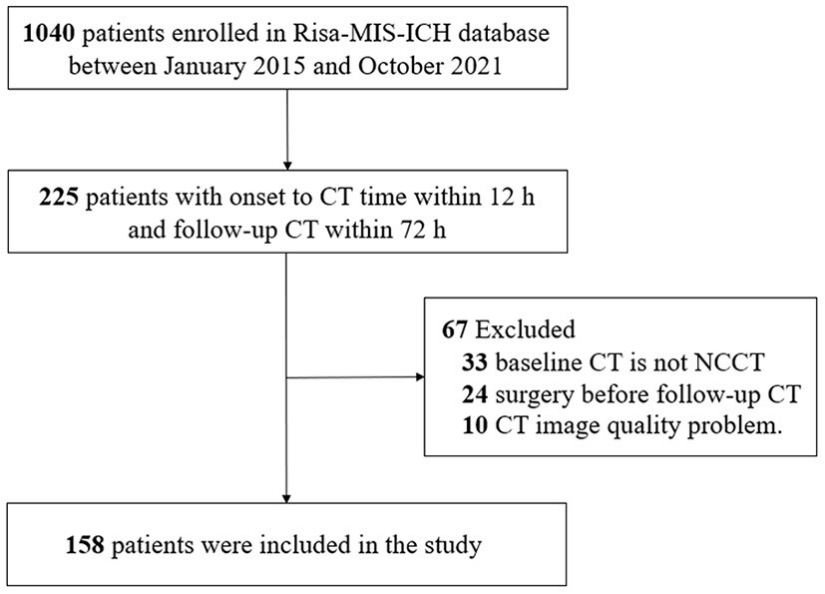
Flowchart of patients included in the study. CT, computed tomography; NCCT, non-contrast computed tomography; Risa-MIS-ICH, Risk Stratification and Minimally Invasive Surgery in Acute Intracerebral Hemorrhage.

This study enrolled a total of 158 patients with ICH (Figure 2). There was no significant difference in patient demographics between included and excluded patients (Supplementary Table 1); 31 (19.6 %) patients were identified as having hematoma growth. The mean age of the patients (±standard deviation) was 61.01 ± 2.5 years, with 128 (81.1%) male patients; 110 (69.6%) patients had hypertension and 29 (18.3%) had diabetes mellitus. Table 1 summarizes the baseline clinical and radiological characteristics of patients with and without hematoma growth. Statistical descriptions of ICH volume, mean HUs of hematoma, standard HUs of hematoma, and CV HUs of hematoma are shown in Supplementary Table 2. Patients with early hematoma growth had a shorter time to baseline CT (*P* = 0.101), a smaller mean HU of hematoma (*P* < 0.001), a larger CV of hematoma HUs (*P* < 0.001) and were more likely to have diabetes mellitus (*P* = 0.045), a black hole sign (*P* = 0.045), and heterogeneous density (*P* < 0.001) than those without early hematoma growth. Age, sex, hypertension, oral anticoagulants, oral antiplatelet drugs, admission systolic blood pressure (SBP), baseline Glasgow Coma Scale (GCS) score, deep ICH, relevant laboratory indicators, swirl sign, baseline ICH volume, and standard HU of hematoma did not differ significantly between patients with and without hematoma growth.

**Table 1:** Clinical and radiological baseline features of individuals with and without hematoma growth.

**Characteristics**	**Total** **(*n* = 158)**	**Hematoma growth**		***P*-value**
		**Yes** **(*n* = 31)**	**No** **(*n* = 127)**	
Age, in years, mean (SD)	61.0 (12.5)	57.7 (12.5)	61.8 (12.4)	0.106
Male Sex, *n* (%)	128 (81.0%)	28 (90.3%)	100 (78.7%)	0.140
Hypertension, *n* (%)	110 (69.6%)	20 (64.5%)	90 (70.9%)	0.491
Diabetes mellitus, *n* (%)	29 (18.3%)	20 (64.5%)	9 (7.1%)	< 0.001
Oral anticoagulants, *n* (%)	3 (1.9%)	1 (3.2%)	2 (1.6%)	0.546
Oral antiplatelet drugs, *n* (%)	4 (2.5%)	2 (6.5%)	2 (1.6%)	0.121
Admission SBP, mmHg (SD)	163.6 (27.7)	165.1 (30.5)	163.3 (27.1)	0.747
GCS score, median (IQR)	13 (10–15)	14 (10–15)	13 (10–15)	0.542
Deep ICH, *n* (%)	130 (82.3%)	28 (90.3%)	102 (80.3%)	0.191
**Density category**				< 0.001
Homogeneous, *n* (%)	142 (89.9%)	21 (67.7%)	121 (95.3%)	
Heterogeneous, *n* (%)	16 (10.1%)	10 (32.3%)	6 (4.7%)	
WBC, in 10^9^/L, mean (SD)	9.5 (3.3)	8.7 (4.4)	9.7 (3.0)	0.142
HGB, in g/L, mean (SD)	142.3 (18.0)	141.1 (19.4)	142.6 (17.7)	0.676
PLT, in 10^9^/L, mean (SD)	212.6 (63.7)	200.1 (78.4)	215.6 (59.5)	0.223
GLU, in mmol/L, mean (SD)	7.4 (3.4)	8.3 (4.8)	7.2 (2.9)	0.248
Ca, in mmol/L, mean (SD)	2.2 (0.1)	2.2 (0.2)	2.2 (0.1)	0.911
Swirl sign, *n* (%)	38 (24.1%)	8 (25.8%)	30 (23.6%)	0.799
Black hole sign, *n* (%)	15 (9.5%)	6 (19.3%)	9 (7.1%)	0.024
Blend sign, *n* (%)	12 (7.6%)	8 (25.8%)	4 (3.1%)	< 0.001
Time to baseline NCCT, in hours, median (IQR)	3.5 (2.0–6.3)	2.1 (1.6–4.5)	3.7 (2.3–6.5)	0.018
Baseline ICH volume, in mL, median (IQR)	24.3 (14.5–36.5)	26.2 (17.8–40.0)	23.8 (14.1–36.4)	0.278
Mean HU of hematoma, in HU, mean (SD)	53.1 (4.1)	56.7 (7.6)	61.6 (4.6)	< 0.001
Standard HU of hematoma, in HU, mean (SD)	10.2 (1.4)	10.6 (1.6)	10.1 (1.4)	0.071
CV HU of hematoma, in %, median (IQR)	17 (15–19)	19 (16–21)	16 (15–18)	< 0.001

*SBP*, systolic blood pressure; *GCS*, Glasgow Coma Scale; *ICH*, intracerebral hemorrhage; *WBC*, white blood cell; *HGB*, hemoglobin; PLT, platelet; *GLU*, blood glucose; *Ca*, serum calcium; *CT*, computed tomography; HU, Hounsfield unit; *CV*, coefficient of variation; *SD*, standard deviation; *IQR*, interquartile range.

### Analysis of risk factors for hematoma growth

Diabetes mellitus, heterogeneous density, black hole sign, blend sign, mean HUs, and CV of hematoma HUs were all linked with hematoma growth in univariate logistic regression (Table 2). Univariate logistic analysis factors that were significant were retained for the multivariate logistic model. The multivariate analysis revealed that time to baseline CT, heterogeneous density, and the CV of hematoma HUs were significant predictors of hematoma growth (Table 3).

**Table 2:** Univariate analysis of predictors for early hematoma growth.

**Variable**	**Odds Ratio**	**95% Confidence Interval**	***P-*value**
Age	0.973	0.941–1.006	0.108
Sex	0.397	0.112–1.405	0.152
Hypertension	0.747	0.326–1.713	0.492
Diabetes mellitus	3.147	1.027–9.639	0.045
Oral anticoagulants	2.083	0.183–23.743	0.554
Oral antiplatelet drugs	2.851	0.455–17.847	0.263
Admission SBP	1.002	0.988–1.017	0.745
Baseline GCS score	1.011	0.879–1.164	0.876
Lobar ICH	0.785	0.323–1.908	0.593
Deep ICH	2.288	0.643–8.133	0.201
WBC	0.906	0.795–1.033	0.142
HGB	0.995	0.974–1.017	0.674
PLT	0.996	0.990–1.002	0.223
GLU	1.082	0.977–1.199	0.129
Ca	0.918	0.061–13.822	0.951
Density heterogeneity	9.603	3.155–29.231	< 0.001
Swirl sign	1.125	0.456–2.774	0.799
Black hole sign	3.147	1.027–9.639	0.045
Blend sign	10.696	2.973–38.474	< 0.001
Time to baseline CT	0.878	0.751–1.026	0.101
Baseline ICH volume	1.016	0.992–1.041	0.199
Mean HU of hematoma	0.829	0.758–0.906	< 0.001
Standard HU of hematoma	1.288	0.976–1.699	0.074
CV HU of hematoma	1.452	1.219–1.729	< 0.001

*SBP*, systolic blood pressure; *GCS*, Glasgow Coma Scale; *ICH*, intracerebral hemorrhage; *WBC*, white blood cell; *HGB*, hemoglobin; *PLT*, platelet; *GLU*, blood glucose; *Ca*, serum calcium; *CT*, computed tomography; *HU*, Hounsfield unit; *CV*, coefficient of variation.

**Table 3:** Multivariate analysis of predictors for early hematoma growth.

**Variable**	**Odds ratio**	**95% Confidence interval**	***P-*value**
Diabetes mellitus	3.476	0.864–13.984	0.079
Heterogeneity	5.950	1.228–28.828	0.027
Black hole sign	0.499	0.097–2.568	0.406
Blend sign	2.159	0.432–10.795	0.349
Time to baseline CT	0.824	0.686–0.991	0.040
Baseline ICH volume	1.012	0.980–1.045	0.466
Mean HU of hematoma	0.892	0.795–1.001	0.052
CV HU of hematoma	1.301	1.047–1.617	0.018

*CT*, computed tomography; *ICH*, intracerebral hemorrhage; *HU*, Hounsfield unit; *CV*, coefficient of variation.

### ROC analysis determines the critical value of CV of hematoma HU value

**Figure 3:**
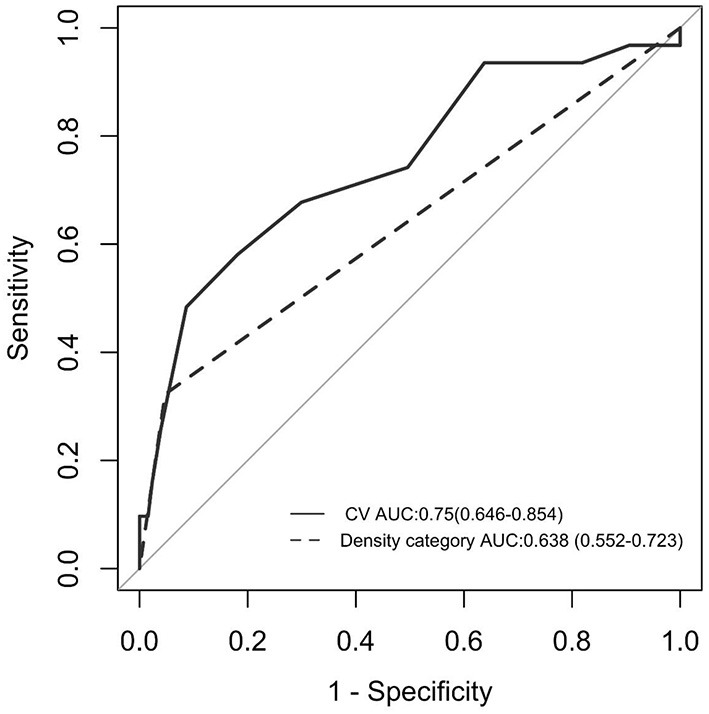
ROC curve analysis between the CV of hematoma HU and early hematoma growth. AUC was 0.750, and the cutoff point was 18% (solid line). ROC curve analysis between “heterogeneous” in the density category and early hematoma growth. AUC was 0.638 (dotted line). AUC, area under the curve; ROC curve, receiver operating characteristic curve; HU, Hounsfield unit; CV, coefficient of variation.

In comparison to heterogeneous density (area under the curve = 0.750), the CV of hematoma HU (area under the curve = 0.638) has a significantly stronger predictive value (Figure 3). The optimum cutoff value representing the CV of hematoma HU value for predicting hematoma growth was 18%, with a specificity of 81.9% and a sensitivity of 58.1%.

## Discussion

Our study revealed that heterogeneous density of hematoma was a significant predictor of hematoma growth. The quantitative heterogeneity of hematomas as characterized by the CV of hematoma HUs was more predictive of hematoma growth than the traditional qualitative heterogeneity score. Diabetes mellitus, the black hole sign, a shorter time to baseline CT, and a smaller mean HU of hematoma were also found to be related to hematoma growth.

The reason for heterogeneity on NCCT is unclear. We postulate that this could be a sign of either persistent bleeding or local coagulopathy, and the available explanations from previous studies are given in the following text. A hematoma develops following the rupture of a brain vessel (23). In the early stages of intracerebral hemorrhage, a hematoma is a heterogeneous mass composed of different blood cells, platelet thrombus, and protein-rich plasma with a relatively high density (24). Due to thrombus contraction and deposition of cell components, low-attenuation plasma is extruded, resulting in a rise in hematoma density (8). Hematoma growth may be cascaded, with increasing evidence supporting the notion of secondary shear hemorrhage with several ruptured vessels surrounding the first hematoma (8, 22). Fresh blood coexists with a subacute blood clot in this model, and the mature area of early bleeding forms the high-attenuation area of hematoma, while the immature area of late hemorrhage forms the low-attenuation area of hematoma, resulting in hematoma heterogeneity (25).

The presence of active contrast extravasation within a hematoma is referred to as a CTA spot sign, and it is frequently used to forecast hematoma growth (2, 26). The frequency of CTA spot signs was found to be inversely proportional to the time of the beginning of cerebral bleeding, and the positive predictive value of speckle signs for substantial hematoma expansion declined as CTA time increased (27). Patients with hematoma growth have a shorter time to baseline CT in our study and in many other studies (13, 24). We speculate that heterogeneity in hematomas is synonymous with the CTA spot sign, which may signify early persistent bleeding.

In comparison to the CTA spot sign, the NCCT is easier to obtain. As a result, NCCT markers have been routinely employed in clinical practice to predict hematoma growth. NCCT markers are classified into two groups based on their shape and density (17). The swirl sign, black hole sign, density heterogeneity scale, hypodensities, and blend sign all indicate hematoma density heterogeneity directly or indirectly (8). These markers have been demonstrated to be predictive of hematoma growth (11–14, 22). However, the current scoring methods lack standardization (17), and the degree of heterogeneity cannot describe the heterogeneity of hematoma (14), which limits the clinical application. In our study, the entire hematoma was considered the region of interest, and the quantitative hematoma heterogeneity index was obtained through automated segmentation tools. Unlike the NCCT marker, the CV HU of hematoma is objective and quantifiable.

The clinically relevant findings of our study are as follows: First, we identified a quantifiable objective predictor of hematoma growth, contrary to other NCCT markers that are subjective. We believe that our predictor may guide the stratification of hematoma risk. Second, our findings have translational potential for clinical applications. For example, our technology makes it possible to create relevant software that can be used to assimilate a large amount of data and establish predictive models *via* machine learning, thereby allowing for automatic recognition and segmentation of hematomas. Clinicians would be able to import the imaging data to extract critical hematoma characteristics such as hematoma volume and CV of hematoma HUs; it can also be incorporated into the imaging workstation as a useful tool for the radiologist. Further large-scale randomized control trials are necessary to further validate our findings and to inform policy guidelines to implement this promising idea of an open-source, freely available software.

This study has certain limitations. First, this is a retrospective analysis with a small sample size; therefore, our findings require further confirmation using the entire data from the Risa-MIS-ICH prospective trial. Second, we included patients with a 12-h baseline CT in our study, as opposed to patients with a shorter baseline CT, which may have resulted in missed cases of possible hematoma growth. Third, with an increase in sample size, the optimal cutoff representing the CV of the hematoma HU value for predicting hematoma growth may change. Finally, the segmentation and processing of hematoma require specialized software, which may be difficult to obtain in some hospitals and institutions.

In conclusion, our study established that the heterogeneity of hematomas may be a predictor of early hematoma growth in patients with ICH. Moreover, the quantitative hematoma heterogeneity index utilized in this study has a significantly greater predictive value than that of the conventionally used heterogeneous density markers on NCCT. At the next stage, the RIS-MIS-ICH project will validate the research findings using prospective multicenter large-sample size data.

## Data availability statement

The raw data supporting the conclusions of this article will be made available by the authors, without undue reservation.

## Ethics statement

The studies involving human participants were reviewed and approved by the Ethics Committee of Fujian Medical University's First Affiliated Hospital (Ethical Approval Number: MRCTA, ECFAH of FMU [2018] 082-1). Written informed consent from the patients/participants or patients/participants' legal guardian/next of kin was not required to participate in this study in accordance with the national legislation and the institutional requirements.

## Author contributions

MZ, WH, SH, and FL: acquisition of data and critical revision of the manuscript for intellectual content. QH, YZ, ZG, LC, and GY: study supervision. RC, WF, DW, and YL: study concept and design. SH and SW: guidance on statistics. DK and LY: analysis and interpretation of data and study supervision. All authors have reviewed the final version of the manuscript.

## Funding

This study was supported by the project on research and application of effective intervention techniques for a high-risk population of stroke from the National Health and Family Planning Commission in China (GN-2018R002) and the National Cerebrovascular and Nervous System Difficult Diseases Diagnosis and Treatment Capacity Improvement Project, Fujian Province High-level Neuromedical Center Construction Fund (Grant No: HLNCC-FJFY-003), as well as Fujian Province Science and Technology Innovation Joint Fund Project (2019Y9118).

## Conflict of interest

The authors declare that the research was conducted in the absence of any commercial or financial relationships that could be construed as a potential conflict of interest.

## Publisher's note

All claims expressed in this article are solely those of the authors and do not necessarily represent those of their affiliated organizations, or those of the publisher, the editors and the reviewers. Any product that may be evaluated in this article, or claim that may be made by its manufacturer, is not guaranteed or endorsed by the publisher.
