# Supplementary material for: Quantitative hematoma heterogeneity associated with hematoma growth in patients with early intracerebral hemorrhage
Source: Front Neurol. 2022 Oct 21;13:999223. doi: 10.3389/fneur.2022.999223 (PMC9634162; doi:10.3389/fneur.2022.999223)

Supplemental Image 1. Hematomas on the axial section were identified layer by layer using a semi-automatic edge detection method in iPlan Cranial 3.0 of Brainlab. The red line is the edge of the identified hematoma.

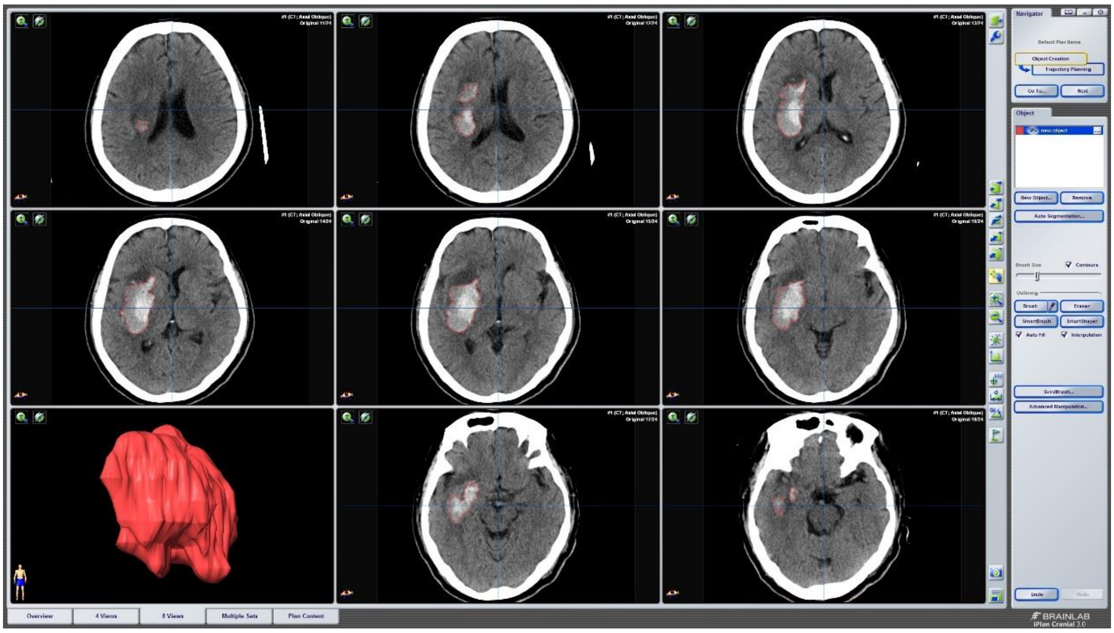

Supplemental Image 2. The region of interest (ROI) including all hematoma was processed to obtain the histogram of Hounsfield units (HU) (lower right corner) using the auto segmentation tool. The left HU shows the hematoma's lowest density and the right HU has its highest density.

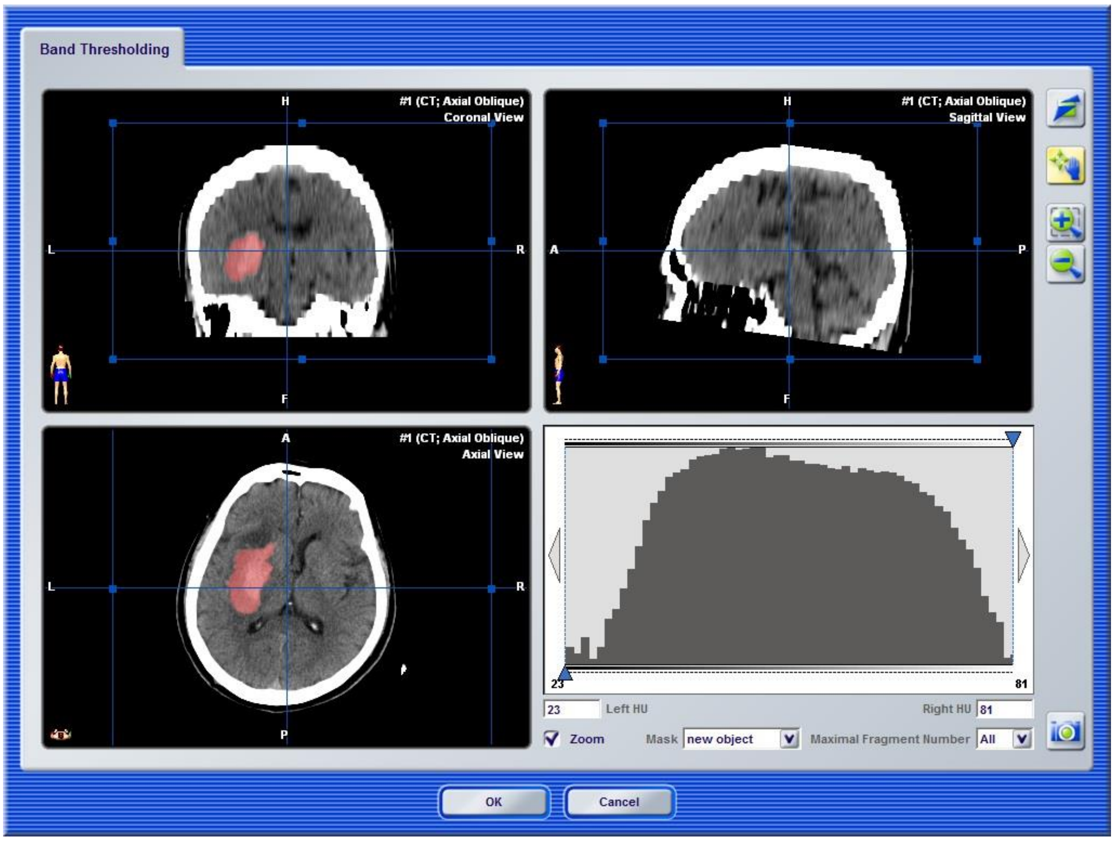

Supplemental Image 3. The region of interest (ROI) which included the entire hematoma was processed to obtain hematoma-related parameters, such as hematoma volume, Mean Housfield units (HU) of hematoma, and standard HU of hematoma.

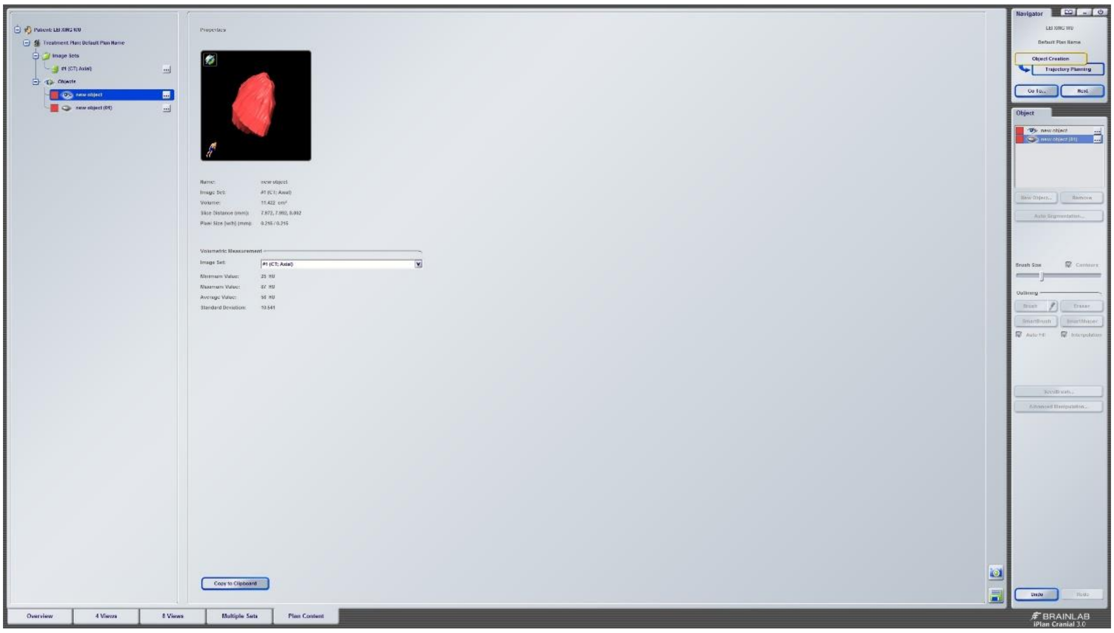

Supplement: Supplementary file 1 [file Data_Sheet_1.pdf]
