# Supplementary material for: Quantitative hematoma heterogeneity associated with hematoma growth in patients with early intracerebral hemorrhage
Source: Front Neurol. 2022 Oct 21;13:999223. doi: 10.3389/fneur.2022.999223 (PMC9634162; doi:10.3389/fneur.2022.999223)
Supplement: Supplementary file 2 [file Table_1.doc]

**Supplementary Table 1** Demographic and ICH features of patients included and excluded.

SBP, Systolic blood pressure; GCS, Glasgow Coma Scale; ICH, Intracerebral hemorrhage.

| **Characteristics** | | | **Included patients**  **(n=158)** | | **Excluded patients**  **(n=882)** | ***P*-value** |
| --- | --- | --- | --- | --- | --- | --- |
| Age, in years, mean (SD) | | | | 61.0 (12.5) | 60.8 (12.6) | 0.857 |
| Male Sex, n (%) | | 128 (81.0%) | | | 724 (82.1%) | 0.773 |
| Hypertension, n (%) | | 110 (69.6%) | | | 625 (70.9) | 0.752 |
| Diabetes mellitus, n (%) | | 29 (18.3%) | | | 123 (13.9) | 0.149 |
| Oral anticoagulants, n (%) | | 3 (1.9%) | | | 9 (1.0%) | 0.341 |
| Oral antiplatelet drugs, n (%) | | 4 (2.5%) | | | 29 (3.3%) | 0.617 |
| Admission SBP, mmHg (SD) | | 163.6 (27.7) | | | 162.1 (25.4) | 0.506 |
| GCS score, median (IQR) | | 13 (10-15) | | | 14 (11-15) | 0.144 |
| Deep ICH, n (%) | 130 (82.3%) | | | | 739 (83.8%) | 0.638 |
