# Supplementary material for: Quantitative hematoma heterogeneity associated with hematoma growth in patients with early intracerebral hemorrhage
Source: Front Neurol. 2022 Oct 21;13:999223. doi: 10.3389/fneur.2022.999223 (PMC9634162; doi:10.3389/fneur.2022.999223)
Supplement: Supplementary file 3 [file Table_2.doc]

**Supplementary Table 2.** The statistical description of the character associated with the hematoma

| Statistical variables | Hematoma Growth  (n=31) | | | | Without Hematoma Growth  (n=31) | | | |
| --- | --- | --- | --- | --- | --- | --- | --- | --- |
|  | ICH volume, in mL | Mean HU of hematoma, in HU | Standard HU of hematoma, in HU | CV HU of hematoma, in % | ICH volume, in mL | Mean HU of hematoma, in HU | Standard HU of hematoma, in HU | CV HU of hematoma, in % |
| AVG | 29.9 | 56.7 | 10.6 | 19.0 | 25.9 | 61.6 | 10.1 | 16.5 |
| MED | 26.2 | 55.0 | 10.5 | 19 | 23.8 | 62.0 | 10.1 | 16 |
| SD | 18.2 | 7.6 | 1.6 | 3.3 | 14.7 | 4.6 | 1.4 | 2.3 |
| MIN | 5.0 | 46 | 8.5 | 11 | 5.4 | 50 | 6.8 | 12 |
| MAX | 80.8 | 88 | 15.6 | 27 | 80.8 | 74 | 14.1 | 23 |

ICH, Intracerebral hemorrhage; HU, Hounsfield units; CV, Coefficient of variation; AVG, Average; MED, Median; SD, Standard deviation; MIN, Minimum values; MAX, Maximum value.
